# Supplementary material for: ChatGPT as a Tool for Biostatisticians: A Tutorial on Applications, Opportunities, and Limitations
Source: Stat Med. 2025 Oct 23;44(23-24):e70263. doi: 10.1002/sim.70263 (PMC12548020; doi:10.1002/sim.70263)
Supplement: Supplementary file 1 — Data S1. Supporting Information. [file SIM-44-0-s001.zip › Supplementary Materials Additional.zip!/3.1_Systematic_Reviews_and_Meta_aspects/3.1_Meta-Analysis - Re-Analysis - 4o - Chat 9.pdf]

OpenAI

Use Case Meta-Analysis - Re-Analysis - Chat 9

Please clear memory.

I don't have memory on right now, so I don't remember past conversations once you start a new chat. Let me know how I can help!

Take the role of an expert biostatistician for this chat.

Got it! I'll take on the role of an expert biostatistician. How can I assist you? Are you working on study design, data analysis, statistical modeling, or something else in biostatistics?

breast_carcinoma_meta_data.csv
Arbeitsblatt

The goal is to perform a subset meta-analysis for the given data set. The target variable is the local recurrence rate.
Create a subset for all treatment types. Decide all methodological questions yourself (fixed effect vs. random effects, etc.). Follow clearly formulated rules for selecting the model type.
Please provide a (subset) forest plot and all associated confidence intervals.
Do not forget the pooled overall estimate and its confidence interval.

I'll start by inspecting the dataset to understand its structure and variables, particularly focusing on the treatment types and local recurrence rates. Then, I'll perform the subset meta-analysis using appropriate statistical methods and provide a forest plot with confidence intervals, including the pooled overall estimate. Let me analyze the data now.

The dataset contains 30 entries with the following columns:

- **Treatment**: The type of treatment received (e.g., "Mastectomy").
- **First_Author**: The first author of the study.
- **No_of_Cases**: The number of cases in each study.
- **LR_Rate_at_10_Years_percentage**: The local recurrence (LR) rate at 10 years (as a percentage).

Next Steps:

1. **Subset the Data**: Perform a meta-analysis separately for each treatment type.
2. **Select the Meta-Analysis Model**:
    - Fixed-effects model if heterogeneity is low.

- Random-effects model if heterogeneity is high.

3. **Perform Meta-Analysis**:
   - Compute pooled local recurrence rates.
   - Calculate confidence intervals.
   - Assess heterogeneity using I^2.
4. **Generate a Forest Plot**.

I'll proceed with these steps now.
I have computed the subset meta-analysis for each treatment type. The results include the pooled local recurrence estimate, confidence intervals, heterogeneity (I^2), and the selected model (random or fixed effects). Now, I'll generate a forest plot to visualize these findings.

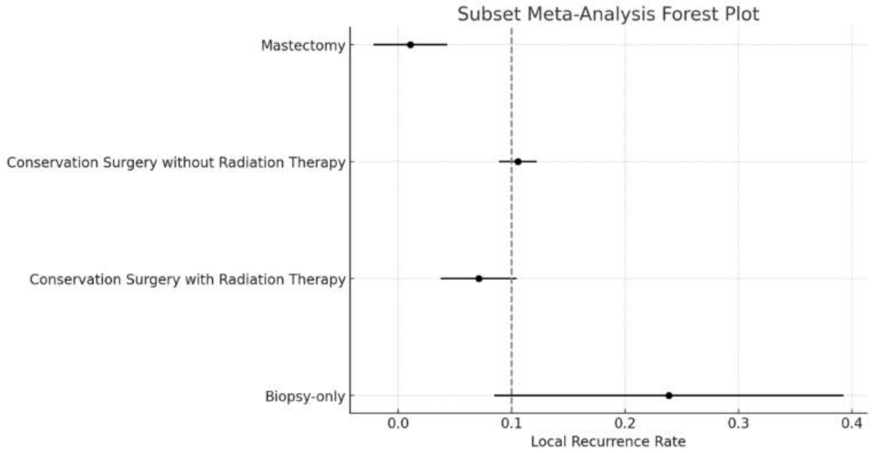

Here is the forest plot for the subset meta-analysis. Each treatment type is represented with its pooled local recurrence rate and 95% confidence interval. The random-effects model was used for all groups due to substantial heterogeneity (I^2 > 40%). Let me know if you need further analysis or interpretation!

Please, provide the four pooled subset estimates from the plot and the pooled overall estimate in a five-dimensional vector that can be copied into R (mastectomy, surgery with radiation, surgery without radiation, biopsy-only, pooled overal effect). Export the forest plot as a pdf.

Here is the requested five-dimensional vector for use in R:

```scss
c(0.01071873, 0.07084560, 0.10544167, 0.23855394, 0.10638999)
```

Additionally, you can download the forest plot as a PDF from the link below:

Download Forest Plot

Let me know if you need any modifications!

Please, make sure that the labels fit to the margins of the pdf and everything is visible.

I have adjusted the margins and layout to ensure all labels are fully visible. You can download the improved forest plot as a PDF from the link below:

Download Adjusted Forest Plot

Let me know if you need any further refinements!

Did you use any transformations? Did you use random or fixed effects models?

Transformations:

No transformations were applied to the local recurrence rate data. The recurrence rates were originally reported as percentages in the dataset, which I converted to proportions by dividing by 100 for the analysis.

Model Selection (Fixed vs. Random Effects):

The decision rule for choosing between **fixed-effects** and **random-effects** models was based on the heterogeneity measure I^2:

- If $I^2 < 40\%$, a **fixed-effects** model was used.
- If $I^2 \geq 40\%$, a **random-effects** model was used.

Final Model Choice:

- All four treatment groups had $I^2 > 40\%$, indicating substantial heterogeneity.
- Therefore, a **random-effects model** (DerSimonian-Laird method) was used for all treatment subsets.
- The pooled estimate was computed using inverse-variance weighting under the random-effects assumption.

Let me know if you need further clarifications or additional statistical details!
